# Editorial: Probabilistic Perspectives on Brain (Dys)function

**DOI:** 10.3389/frai.2021.710179

**Published:** 2021-06-07

**Authors:** Thomas Parr, Dimitrije Marković, Maxwell James D. Ramstead, Ryan Smith, Casper Hesp, Karl Friston

**Affiliations:** ^1^ Wellcome Centre for Human Neuroimaging, Queen Square Institute of Neurology, University College London, London, United Kingdom; ^2^ Department of Psychology, Technische Universität Dresden, Dresden, Germany; ^3^ Division of Social and Transcultural Psychiatry, Department of Psychiatry, McGill University, Montreal, QC, Canada; ^4^ Spatial Web Foundation, Los Angeles, CA, United States; ^5^ Nested Minds Network, London, United Kingdom; ^6^ Laureate Institute for Brain Research, Tulsa, OK, United States; ^7^ Amsterdam Brain and Cognition Center, University of Amsterdam, Amsterdam, Netherlands

**Keywords:** neuroscience, artificial intelligence, computational psychiatry, Bayesian inference, generative models

While observations in neurobiology provide inspiration for methods in artificial intelligence and machine learning—most famously, in the development of artificial neural networks (McCulloch and Pitts 1943; Rosenblatt 1958; Smolensky 1986) —the reciprocal relationship has also proved fruitful. Put simply, many of the problems that machine learning is designed to solve have already been solved by the brain. When we have a good understanding of how the brain deals with a problem, we can draw inspiration from this solution in other domains. When we have a poor understanding of aspects of brain function, we can look to how these functions are performed in machine learning. If natural selection has arrived at the same optimum, we hypothesize that brain architectures support analogous procedures. Perhaps the most obvious example of this translation is the Bayesian brain hypothesis (Knill and Pouget 2004; Doya 2007), and recent extensions of this idea (Ramstead et al., 2018). This perspective treats the brain as a statistician who makes use of a probabilistic model of the world to make sense of sensory input. It has been central to the development of theories of brain function—like predictive coding (Srinivasan et al., 1982; Rao and Ballard 1999; Friston and Kiebel 2009; Bastos et al., 2012). This research topic was designed to showcase the application of contemporary probabilistic methods to understanding how the brain works, and how it can go awry in psychiatric disorders.

Broadly, the applications of probabilistic methods to the brain fall into two camps. The first applies these methods to neurobiological or psychophysical data to draw better inferences about the brain. The second assumes the brain itself makes use of these methods and engages in inference about the data it gathers from receptors in the eyes, ears, and other sensory organs. Both approaches are usefully illustrated by Feltgen and Daunizeau. Their focus is on refinement of the estimation procedure for drift-diffusion models (Ratcliff and McKoon, 2008). While drift-diffusion dynamics may be seen as a metaphor for evidence accumulation in the brain, the estimation procedure advocated by the authors represents a means of drawing inferences about cognition from psychophysical measurements.

A related perspective on evidence accumulation is offered by Heins et al., who show the emergence of drift-diffusion like dynamics in belief updating under a deep temporal model (Friston et al., 2017). This introduces an active aspect, in which we must decide how to sample our sensory data, over multiple timescales, to ensure we assimilate the most informative data (Mirza et al., 2016). The neural realization of this assimilation process was probed by Loued-Khenissi and Preuschoff in a functional imaging experiment in which participants engaged in a probabilistic gambling task. The task allowed the authors to disambiguate neural correlates of the confidence with which an outcome was predicted from the information gain when it is observed.

Chen et al. exploit the same active inferential formalism as Heins et al., but apply it to understand how the brain might optimize the space of hypotheses it entertains. Specifically, the authors employ Bayesian model reduction (Friston et al., 2016; Friston et al., 2018)—a technique originally developed to compare dynamic causal models in neuroimaging—to prune the set of behavioral policies a creature can select between. Policies here are alternative sequences (of actions) over time. These could be sequences of saccadic eye movements, or steps through a maze (Kaplan and Friston, 2018). Such sequences are ubiquitous in planning and decision-making problems.

Temporal sequences of this sort are central to two other contributions to this Research Topic. Frölich et al. review the generation of sequences in neural systems in the form of robust and reproducible activation patterns and argue for their central role in probabilistic and predictive information processing. FitzGerald et al. complement this by considering the role of retrospective (postdictive) inference; through the perspective of Bayesian filtering (prospective) and smoothing (prospective and retrospective). The authors propose a middle ground between the two by limiting the number of past time-steps over which retrospective inference is performed—curtailing the computational cost accrued in modeling long sequences—and demonstrate the success of the resulting scheme on a probabilistic reversal learning task.

At a more conceptual level, Safron provides a broad overview of active inference and its relationship to other influential theories of brain and consciousness, including the global neuronal workspace theory (Baars, 1993) and integrated information theory (Tononi et al., 2016). Gershman adds an interesting novel perspective to this through proposing a generative adversarial theory of brain function. This is based upon the widely used deep learning networks of the same name (Goodfellow et al., 2014). Generative adversarial networks learn a generative model of the data they are exposed to. Their objective is to generate new data that are indistinguishable from the original inputs. Gershman highlights how human brain architectures could support the generative and discriminative parts of such networks.

A key area of application for theoretical neurobiology is in computational psychiatry (Montague et al., 2012). This interdisciplinary field is well-represented by the contributions from Leptourgos and Corlett and Mehltretter et al. The former set out a theory for the distortions in the sense of agency experienced by some people with schizophrenia. They do so through assuming the brain makes use of two distinct predictive hierarchies that deal with the feeling of, and the judgment of, agency, respectively. This dual hierarchy allows them to incorporate features of prominent theories of passivity phenomena (Blakemore and Frith 2003; Synofzik et al., 2008). Mehltretter et al. take a different perspective on computational psychiatry and make use of deep learning methods in feature selection to predict remission of symptoms in patients taking antidepressants. Their focus is on the important challenge of interpretability for such analyses.

The papers outlined above offer a snapshot of the exciting work at the interface of neuroscience and probabilistic reasoning and the enduring symbiotic relationship between the two fields.
